# Development of novel real-time PCR methodology for quantification of *COL11A1* mRNA variants and evaluation in breast cancer tissue specimens

**DOI:** 10.1186/s12885-015-1725-8

**Published:** 2015-10-14

**Authors:** Makrina Karaglani, Ioannis Toumpoulis, Nikolaos Goutas, Nikoleta Poumpouridou, Dimitrios Vlachodimitropoulos, Spyridon Vasilaros, Ioannis Rizos, Christos Kroupis

**Affiliations:** 1 Department of Clinical Biochemistry and Molecular Diagnostics, Attikon University General Hospital, University of Athens Medical School, Rimini 1 St., Haidari, 12462 Greece; 2 Department of Cardiothoracic Surgery, Attikon University General Hospital, University of Athens Medical School, Athens, Greece; 3 Pathologic Anatomy Laboratory, Evgenidio Hospital, University of Athens Medical School, Athens, Greece; 4 Prolipsis Breast Cancer Clinic, Athens, Greece; 5 Department of Cardiology, Attikon University General Hospital, University of Athens Medical School, Athens, Greece

**Keywords:** *COL11A1*, Variants, Breast cancer, Real-time qPCR

## Abstract

**Background:**

Collagen XI is a key structural component of the extracellular matrix and consists of three alpha chains. One of these chains, the α1 (XI), is encoded by the *COL11A1* gene and is transcribed to four different variants at least (A, B, C and E) that differ in the propensity to N-terminal domain proteolysis and potentially in the way the extracellular matrix is arranged. This could affect the ability of tumor cells to invade the remodeled stroma and metastasize. No study in the literature has so far investigated the expression of these four variants in breast cancer nor does a method for their accurate quantitative detection exist.

**Methods:**

We developed a conventional PCR for the general detection of the general *COL11A1* transcript and real-time qPCR methodologies with dual hybridization probes in the LightCycler platform for the quantitative determination of the variants. Data from 90 breast cancer tissues with known histopathological features were collected.

**Results:**

The general *COL11A1* transcript was detected in all samples. The developed methodologies for each variant were rapid as well as reproducible, sensitive and specific. Variant A was detected in 30 samples (33 %) and variant E in 62 samples (69 %). Variants B and C were not detected at all. A statistically significant correlation was observed between the presence of variant E and lymph nodes involvement (*p* = 0.037) and metastasis (*p* = 0.041).

**Conclusions:**

With the newly developed tools, the possibility of inclusion of *COL11A1* variants as prognostic biomarkers in emerging multiparameter technologies examining tissue RNA expression should be further explored.

**Electronic supplementary material:**

The online version of this article (doi:10.1186/s12885-015-1725-8) contains supplementary material, which is available to authorized users.

## Background

Breast cancer is the most frequent cancer among women both in more and in less developed World regions and the second most commonly occurring form of cancer globally when both sexes are accounted [1]. The search for new prognostic and predictive tissue biomarkers is considered imperative for improving classification of this common type of cancer and for avoiding excessive and unnecessary exposure to toxic and ineffective treatments.

One of such biomarkers could be collagen as it is a key structural component of the extracellular matrix (ECM) that also serves as a modulator of diverse signaling pathways. Collagen XI belongs to the minor fibrillar subcategory in the collagen family and it is responsible for the proper conformation of collagen II and the formation of thin fibrils of developing or under remodeling tissues. Its highest expression values have been found in the articular cartilage and vitreous humor [2, 3]. It is a heterotrimeric protein, consisting of three alpha chains (a1, a2 and a3) that are organized into a triple helix formation. Both a1(XI) and a2(XI) chains are unique gene products however, a3(XI) is a an hyperglycolsylated version of the collagen a1(II) chain [4, 5]. The a1(XI) chain is encoded by the gene *COL11A1* located at genomic locus 1p21.1. It is initially synthesized as procollagen XI and then its C and N termini may be cleaved with proteolysis as soon as they are secreted from the cell [6]. The molecule of the a1(XI) chain has a characteristic globular N-terminal domain (NTD) consisting of a variable region and an amino-propeptide (Npp) that seems responsible for the steric hindrance exerted by collagen XI to other molecules in the ECM [7, 8]. Therefore, when collagen a1(XI) protein is overexpressed -as it has been proven in human ascending thoracic aortic aneurysms-, it leads to thinner collagen fibers and decreased tensile strength in the tissue [9].

It has also been demonstrated that expression of collagens alters in neoplasms, a fact that could affect the ability of tumor cells to break through the basal membrane and initiate local or distant metastases [10–12]. *COL11A1* upregulation in tumor tissue versus normal tissue has been demonstrated in gastric cancer [13], non-small cell lung cancer [14, 15], pancreatic cancer [16] and this expression has been associated with metastasis in oral cavity and oropharynx [17], ovarian [18] and lung cancer [15]. In ovarian cancer, it leads to a stromal desmoplastic reaction in cancer-associated fibroblasts, a feature that is associated with the epithelial-to-mesenchymal transition (EMT) phenotype [19]. In a significant study for breast cancer, *COL11A1* is shown to be significantly upregulated in infiltrating tumor lesions compared to their *in situ* compartments and adjacent stroma [20]. In another study though, collagen a1(XI) appears to be downregulated in stroma surrounding breast cancer but also in metastasized tumors [21]. In addition, *COL11A1* is differentially expressed between primary breast cancers that metastasize and their corresponding lymph node sites where its expression seems that is no longer needed [22, 23]. The detection of such quantitative changes in *COL11A1* expression could lead to novel approaches regarding prognostic and/or predictive tools for breast cancer.

**Fig. 1:**
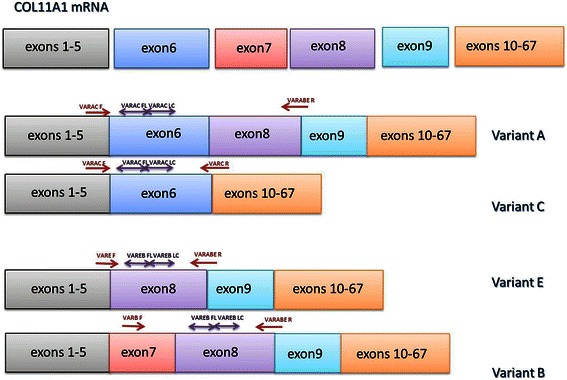
Structure of *COL11A1* splice variants A, B, C and E and approximate location of primers and set of dual probes in respect to each different variant in the design of the novel *COL11A1* assays: variants A and C employ a common set of probes, variants B and E employ a second different common set of probes and a common reverse primer

*COL11A1* gene consists of 67 exons and due to alternative splicing of four exons (6, 7, 8 and 9), there exist possibilities of production of at least eight different variants during its transcription [24–26]. Four different splicing variants of *COL11A1* mRNA termed A, B, C and E, (Fig. 1) have been deposited in GenBank (Table 1) and are known to differ in their propensity for NTD proteolysis [27] and potentially in the way the extracellular matrix is arranged. No study in the literature has so far investigated the expression of the four known variants in breast cancer (as well as cancer in general) nor does a method for their accurate quantitative detection exist.

**Table 1:** GenBank Accession numbers used for the detection of the C*OL11A1* mRNA splice variants and the general transcript and the sizes of the expected real-time PCR products according to our design strategy

*Name*	*GenBank*	*Expected PCR product size*
	*Accession number*	
*Variant Α*	NM_001854	439
*Variant Β*	NM_080629	379
*Variant C*	NM_080630	206
*Variant Ε*	NM_001190709	259
*General transcript*	NG_008033.1	132

In our study we validated novel, specific and sensitive real-time qPCR (quantitative Polymerase Chain Reaction) methodologies for *COL11A1* mRNA variants in the Lightcycler platform and obtained quantitative data for their distribution in breast tumors. Furthermore, we sought to determine whether there is a correlation between differential expression of these *COL11A1* splice variants with tumor histopathological parameters and patient follow-up data in order to explore the possibility of their inclusion as prognostic biomarkers in emerging multiparameter technologies examining tissue RNA expression (analogous to Oncotype, MammaPrint, HOXB13: IL17BR and molecular grade index 8-gene panel, Endopredict and PAM50) [28–32].

## Methods

### Patients

Ninety tissue specimens were collected from the Pathologic Anatomy Laboratory of Evgenidio Hospital from consecutive female breast cancer patients residing mostly in the Athens Metropolitan area during the period 2007–2011. Main criteria were the availability of the material, the presence of >70 % of tumor cells in the frozen section and the written informed consent of the patients (family history was not used as a criterion for inclusion in the study). The study was approved by both bioethics and scientific committees of the Evgenidio Hospital. Most of the specimens originated from lumpectomies and the mean size was 2.0 cm (range: 1.0–5.5 cm). A small part of the resected specimens at surgery was immediately stored in RNAlater (Life Technologies Ambion, USA) for 1–2 days at 4 °C and then stored at −80 °C until total RNA extraction for molecular collagen analysis. The larger part of the resected specimens was embedded in formalin-fixed paraffin blocks and used for histopathological examinations. The majority of the tumors (80 %) were ductal infiltrating carcinomas (the rest lobular mostly, papillary and mucinous) and were classified according to the Bloom-Richardson grading system as grade 1 (3 samples), grade 2 (57 samples) and grade 3 (22 samples). Grades 1 and 2 were grouped together because of the small number of grade 1 tumors. The presence or absence of estrogen and progesterone hormone receptors was investigated with routine immunohistochemistry (IHC) and positivity was defined as a score >1 in IHC. Oncogene *HER2* overexpression was examined with IHC and when the score was 2 in the 0–3 scale, it was further examined with chromogenic in situ hybridization (CISH). Therefore, we were able to dichotomize all samples as being either HER2 negative or positive. Classification into the triple negative breast cancer (TNBC) category was assigned if a tumor was negative for estrogen and progesterone hormone receptors and HER2 overexpression. Lymph node involvement was also noted and the presence of any recurrences or metastasis was recorded for those patients with follow-up data. The characteristics of the 90 tissues and patients with breast cancer are summarized in Table 2.

**Table 2:** Clinical characteristics of the 90 tissue samples from patients with breast cancer

*Variable*	*Value*
*Age Group, n (%)*	
< = 50 years	26 (35.6)
> 50 years	47 (64.4)
*Tumor Size, n (%)*	
≤ 2.0 cm	59 (67.0)
> 2.0 cm	29 (33.0)
*Histopathological Type, n (%)*	
Lobular infiltrating & rest	18 (20.0)
Intraductal infiltrating	72 (80.0)
*Lymph-node Involvement, n (%)*	
Negative (Ν_0_)	55 (67.1)
Positive (Ν_+_)	27 (32.9)
*Metastasis, n (%)*	
Negative	47 (85.5)
Positive	8 (14.5)
*Grade, n (%)*	
Low (1–2)	60 (73.2)
High (3)	22 (26.8)
*Estrogen-receptor Status, n (%)*	
Negative	21 (23.9)
Positive	67 (76.1)
*Progesterone-receptor Status, n (%)*	
Negative	46 (52.3)
Positive	42 (47.7)
*HER2 Overexpression Status, n (%)*	
Negative	72 (81.8)
Positive	16 (18.2)
*TNBC status, n (%)*	
Yes	17 (19.3)
No	71 (80.7)

### Total RNA Isolation

Total RNA was extracted with the use of the NucleoSpin RNA kit (Macherey-Nagel, Germany) after passing the liquid N_2_-snap frozen tissues through special filter columns (shredders) in order to homogenize them and to reduce viscosity. DNA was removed by an in-column recombinant DNase treatment. Total RNA was eluted in RNase-fee water and stored at −80 °C until further use. The absolute measurement of RNA concentration was determined by the Quant-iT RNA Assay kit in the Qubit 1.0 fluorometer (Life Technologies Invitrogen, USA) that employs a dye specific for RNA and not for DNA.

### Complementary DNA Synthesis

cDNA was synthesized from 1 μg of total RNA and random hexamers in a 20 μL total volume, according to the Transcriptor First Strand cDNA Synthesis kit (Roche Applied Science, Switzerland) instructions. It was organized in large batches and appropriate controls were added: a no-RNA blank (RNA^−^) control, a Reverse Transcriptase-negative (RT^−^) control and a 100 ng RNA-positive (RNA^+^) control for Porphobilinogen deaminase (*PBGD*) gene provided by the kit. The cDNA samples were then stored at −20 °C. In order to test the quality and purity of RNA samples, the resulting cDNA was amplified in a control PCR method of the actin reference gene as previously described [33]. cDNA samples that are free of containing genomic DNA produce a unique fragment of 587 base pairs (bp) (and not the additional fragment of 1122 bp if genomic DNA exists). The efficiency of cDNA synthesis was also examined with conventional PCR for the *PBGD* gene with primers provided by the kit: the same intensity of a 151 bp band was obtained each time for the RNA^+^ control (also many tumor cDNA samples were run alongside as an additional control of quality and purity of the RNA samples).

### Conventional PCR for the general *COL11A1* transcript

In order to detect the presence or not of the general *COL11A1* transcript, a simple conventional PCR was developed. Suitable primers were designed, common for all splice variants of *COL11A1* gene in a well conserved region, by using the CLC Free Workbench version 4 software (Qiagen Bioinformatics, Aarhus, Denmark). The primers shown in Table 3 are located in the junction of exons 48/49 and 51, respectively. For each reaction, 1.5 μL of cDNA was placed in a 23.5 μL reaction mixture containing 12.5 μL of BioMix Red DNA polymerase (Bioline, Germany), 1.5 μL of the supplied MgCl_2_ (50 mM), 1 μL of the primers (final concentration: 0.04 pmol/μL) and ddH_2_O. The cycling protocol was consisted of an initial 4-min denaturation step at 94 °C, followed by 40 cycles of denaturation at 94 °C for 30 s, annealing at 57 °C for 30 s, extension at 72 °C for 30 s and a final 5 min extension step at 72 °C. Checking for the proper size of 132 bp was performed with electrophoresis of a 10 μL PCR product on 2 % w/v agarose gel along with MW marker (PCR Marker, New England Biolabs, USA), staining with ethidium bromide and visualization under ultraviolet (UV) light.

**Table 3:** Sequences of primers and probes of *COL11A1* transcript variants

	*Name*	*Oligonucleotide Sequence, 5’-3’*
*Variant A & C Forward Primer*	VARAC F	TGTGAGCATTATAGTCCAGACTGTGA
*Variant E Forward Primer*	VARE F	CAGATAGATGAGGCAAACATCG
*Variant B Forward Primer*	VARB F	AAGAAGATGAGGACAGTGGCTA
*Variant C Reverse Primer*	VARC R	CCATGGCCATTTATCTCCGT
*Variant A, E & B Reverse Primer*	VARAEB R	CATATTCGCCTAAATCTCCATCTAC
*Variant A & C Sensor Probe*	VARAC FL	TCCTCAGTTACAGTGGGTCCCTCTGTTAC-FL
*Variant A & C Anchor Probe*	VARAC LC	LC 640-CTTTCAGCCTCTTTATACTCTGCTTCCCCA
*Variant E & B Sensor Probe*	VAREB FL	GCTCATTTGTCCCAGAAATGCC-FL
*Variant E & B Anchor Probe*	VAREB LC	LC 640-AGGAGCTTCTGTCTGGTAACTTTCCATTGT
*General COL11A1 Forward Primer*	F	AATGGAGCTGATGGACCACA
*General COL11A1 Reverse Primer*	R	TCCTTTGGGACCGCCTAC

### Real-time quantitative PCR methodology for the *COL11A1* variants detection

For the quantification of *COL11A1* transcript variants, suitable pairs of primers and hybridization sets of dual probes (labeled with fluorescein donor and LC-Red 640 acceptor dyes) were designed by aligning all four variants mRNA in the CLC Free Workbench version 4 program in order to select for non-homologous regions for their binding. The choice of the primers was based on the presence or absence of exons 6, 7, 8 and 9 which differs in different variants uniquely. Transcripts A and C employ a common set of dual probes for their detection but different primers; the same strategy is used for B and E transcripts (Fig. 1). The sequences of primers and probes synthesized by TIB MOLBIOL (Germany) are shown in Table 3.

Real-time quantitative PCR was performed with the LightCycler 1.5 platform (Roche Applied Science) in glass capillaries in a total volume of 10 μL. For transcript variant A, 1 μL of the sample cDNA was added to 0.3 μL of the forward primer VARAC F (final concentration: 0.6 pmol/μL), 0.1 μL of the reverse primer VARAEB R (final concentration: 0.2 pmol/μL), 0.6 μL of the probe VARAC FL (final concentration: 0.18 μΜ), 0.6 μL of the probe VARAC LC (final concentration: 0.18 μΜ), 2 μL of 25 mM MgCl_2_ (Roche, final concentration: 5 mM), 1 μL of the LightCycler FastStart DNA Master HybProbe 10× reagent (Roche Applied Science) and ddH_2_O to the final volume (for variant C, the VARC R primer is used instead of VARAEB R). For transcript variant E, 1 μL of the sample cDNA was added to 0.3 μL of the forward primer VARE F (final concentration: 0.6 pmol/μL), 0.1 μL of the reverse primer VARAEB R (final concentration: 0.2 pmol/μL), 0.5 μL of the probe VAREB FL (final concentration: 0.15 μΜ), 0.5 μL of the probe VAREB LC (final concentration: 0.15 μΜ), 1.2 μL of 25 mM MgCl_2_ (Roche, final concentration: 3 mM), 0.6 μL of DMSO, 1 μL of the LightCycler FastStart DNA Master HybProbe 10× reagent and ddH_2_O to the final volume (for variant B, the VARB F primer is used instead of VARE F). All reactions were initiated with a 10-min denaturation at 95 °C and terminated with a 30 s cooling step at 40 °C. The cycling protocol consisted of denaturation step at 95 °C for 10 s, annealing at 52 °C for variant A/50 °C for variant E for 30 s and extension at 72 °C for 30 s and repeated for 42 cycles. In each preparation, alongside the unknown samples, standards, blank samples and positive controls samples (that were confirmed by DNA sequencing analysis) were included. Fluorescence detection was performed at the end of each extension step for 0 s at the F1 channel. For quantification, an external standard curve was obtained by using the transcript variants PCR amplicon standards (prepared as described below) and plotting the log number of copies corresponding to each standard versus the value of their corresponding quantification cycle (Cq). Real-time qPCR products were additionally checked: i) for size and purity by inversion of the glass capillaries and electrophoresis on 2 % w/v agarose gels (the expected PCR product sizes are provided in the last column of Table **1**) and ii) for nucleotide composition. The Sanger DNA sequencing methodology was performed with a PCR product column clean-up (NucleoSpin Gel and PCR Clean-up kit, Macherey-Nagel, Germany) and a cycle sequencing reaction employing the Big Dye 1.1 reagent (Life Technologies Applied Biosystems, USA). The electrophoregrams in the ΑBI Prism 310 Genetic Analyzer were manually base-called with the Chromas Lite 2.01 software (Technelysium Pty, Tewantin, Australia) and compared with the expected sequence with the BLAST tool of PubMed. Also the T_m_’s of the amplicons were determined immediately after amplification, by melting curve analysis performed in the LightCycler. The melting curve protocol included raising the temperature at 95 °C, cooling at 55 °C for 15 s and slow heating to 95 °C at a rate of 0.1 °C/s, during which time fluorescence measurements were continuously collected in the F2 channel and their first derivate (−d(F2)/d*T vs. T*) was used for the determination of T_m_.

To establish specific, sensitive and reproducible real-time quantitative assays, we performed extensive optimization of primers, probes and MgCl_2_ concentrations as well as of the reaction temperatures and cycles. The analytical evaluation of assays was performed with the prepared standards. For each splice variant detected in our samples, a calibration curve was generated from serial dilutions e.g. ranging from 5 × 10^5^ to 5 × 10^1^ copies/μL of variant A and 5 × 10^6^ to 5 × 10^1^ copies/μL of variant E. The reproducibility (calculated as coefficients of variation, CVs), the efficiency of the PCR reaction (expressed as *E* = 10^-1/slope^) and the limit of detection for our assays (defined as the concentration detected in 95 % of trials) were also determined in order to complete the validation file of the novel methodologies with the established MIQE guidelines [34].

### Preparation of the standards

For the development and analytical evaluation of our assays, we generated and used as standards PCR amplicons corresponding to the *COL11A1* splice variants studied. For this reason, a significant amount of the amplicons was produced by many PCR reactions of the same cDNA preparation in a positive sample for each variant. The amplicons were pooled, purified by columns and quantitated by the Quant-iT dsDNA Broad-Range Assay kit (Life Technologies Invitrogen, USA) in the Qubit 1.0 fluorometer. The concentration was converted to copies per microliter by using the Avogadro constant and the product’s molecular weight (number of bases of the PCR product multiplied by the average molecular weight of a pair of nucleic acids, which is 660), as described elsewhere [35]. Then, serial dilutions of the above-quantified stock amplicon solutions were prepared for each variant and kept in aliquots at −20 °C; they were used throughout the study as external standards for the absolute quantification of *COL11A1* transcript variants.

### Normalization

Normalization facilitates experimental problems concerning the inherent variability of RNA level of expression, variability of extraction protocols and presence of inhibitors [36]. In our assay, we ensured that the starting tissue material for RNA extraction had similar initial size and weight (approximately 30 mg) and we performed normalization against the same amount of total RNA (1 μg) that was used for cDNA synthesis in all samples as suggested by previous studies [36–38].

### Statistical analysis

The *COL11A1* variants were analyzed statistically both in a qualitative way (presence or absence of the variant) with either Pearson *χ* ^2^ or Fischer’s exact test and in a quantitative way: the positive samples were divided in two categories (high or low category) depending whether their copies were above or below a certain percentile value of copies (e.g. the 25^th^, 50^th^ or median, the 75^th^) and 2 × 2 cross-tabulations were performed. Also the median copy values of the two low and high categories were compared in each category of the clinicopathological characteristics examined (all divided in two categories as well) with the Mann–Whitney *U* test for continuous variables that are non-normally distributed (as determined with the Kolmogorov-Smirnov test). The Spearman correlation coefficient was used as a measurement of correlation for continuous non-normally distributed variables. Probit statistical analysis was used for estimation of the limit of detection in our novel assays. The association of COL11A1 transcript variants with long-term metastasis was analyzed with the Kaplan-Meier method and survival curves were compared with the log-rank test. For all tests performed, a two-sided p value of <0.05 was considered significant. Data analysis was carried out with the SPSS version 21.0 statistical software package for Windows (IBM - SPSS Inc., USA).

## Results

### Conventional PCR for the general *COL11A1* transcript

All extracted RNAs were of adequate quantity -as measured in the fluorometer- and quality as they produced a single pure actin band in the gels. The general *COL11A1* transcript was detected in all samples (Additional file 1: Figure S1) as revealed from a distinct 132 bp band in all PCR products.

### Development, analytical and clinical evaluation of the real-time qPCR methodology for the *COL11A1* variants detection

Real-time qPCR methodologies were developed adequately, were rapid and specific as it can be seen in Additional file 2: Figures S2 and Additional file 3: Figure S3 when the real-time PCR products from positive cDNA samples were extracted and run on a 2 % w/v agarose gel: variants A and E produced the expected bands at sizes of 439 and 259 bp. Portions of Sanger DNA Sequencing electropherograms of these transcripts A and E are shown in Additional file 4: Figures S4 and Additional file 5: Figure S5 and are aligned fully with the GenBank deposited variant sequences. Variants B and C were not detected in any tumor cDNA sample, therefore no further validation procedures were performed for these two transcripts.

**Fig. 2:**
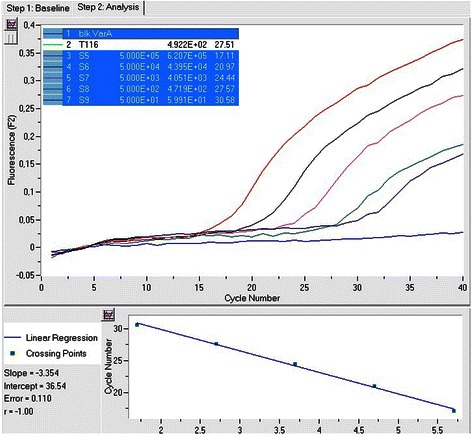
Representative standard curve for the real-time qPCR detection of *COL11A1* variant A: amplicons ranging from 5 × 10^5^-5 × 10^1^ copies A/μl serve as standards, the blue line is the blank of the assay

**Fig. 3:**
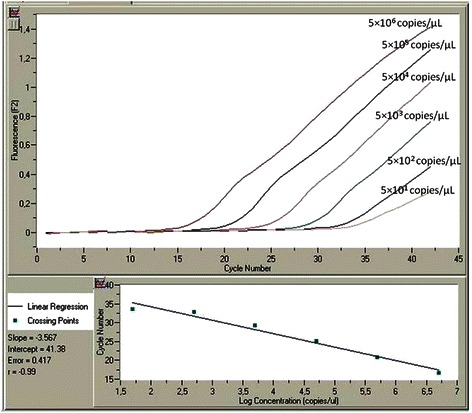
Representative standard curve for the real-time qPCR detection of *COL11A1* variant E: amplicons ranging from 5 × 10^6^-5 × 10^1^ copies E/μl serve as standards

**Fig. 4:**
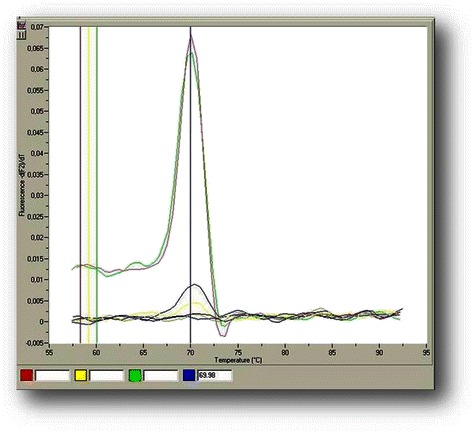
Results from the real-time qPCR assay for *COL11A1* variant A in tumor breast cDNA samples: five positive samples, two negative and a blank (green line)

**Fig. 5:**
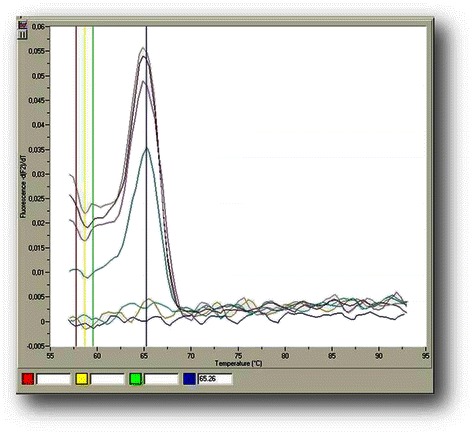
Results from the real-time qPCR assay for *COL11A1* variant E in tumor breast cDNA samples: four positive samples, one negative and a blank (gold line)

The analytical sensitivity and linearity of the proposed *COL11A1* A and E transcript real-time qPCR assays were determined by using the external standards of each variant with known concentrations that were prepared as described above. Our standard curves showed linearity over the entire quantification range (5 × 10^5^ to 5 × 10^1^ variant A copies/μL and 5 × 10^6^ to 5 × 10^1^ variant E copies/μL) while the correlation coefficients were about 0.99 in all cases, indicating a precise log–linear relationship (Figs. 2 and 3). The mean slope and intercept of the standard curve of variant A were −3.22 ± 0.19 and 36.81 ± 0.52 respectively (*n* = 5), while the PCR reaction efficiency was 2.05 ± 0.04 (CV % = 1.97), very close to the ideal value which is 2.00. About variant E, the mean slope and intercept of the standard curve were −3.66 ± 0.34 and 41.80 ± 2.49 respectively (*n* = 5), while the efficiency was 1.88 ± 0.10 (CV % = 5.39). The between-run CV’s for the Cq values of the standards, analyzed in five different experiments over a period of 1 month, ranged from 0.78 to 1.84 % for variant A and from 2.62 to 3.88 % for variant E. The analytical limit of detection as determined from probit statistical analysis was 19 copies/μL for variant A and 16 copies/μL for variant E. The T_m_ from all positive variant A amplicons was calculated to be 69.9 (±1.0) °C, while the corresponding for variant E was 65.3 (±1.2) °C (representative samples in Figs. 4 and 5).

**Fig. 6:**
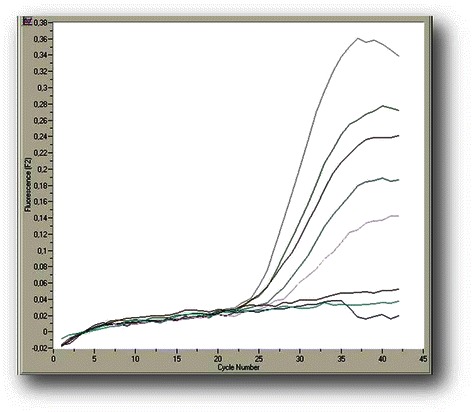
Melting point (Tm) of *COL11A1* variant A amplicon: in this –d(F_2_)/dT *vs.* temperature graph, it is derived from the mean of two strong positive and a weak cDNA sample

**Fig. 7:**
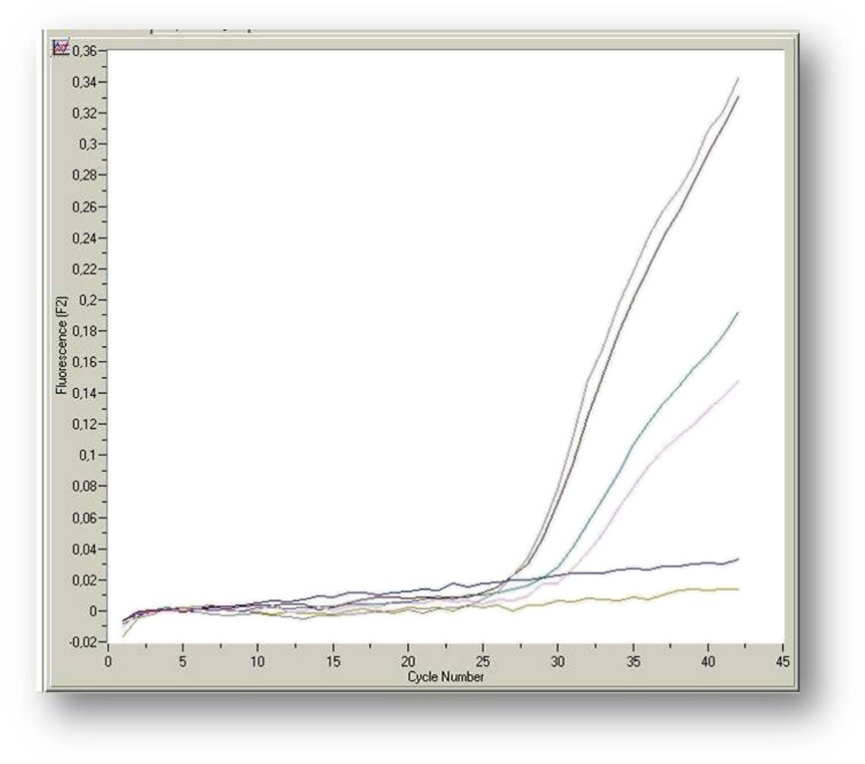
Melting point (Tm) of *COL11A1* variant E amplicon: in this –d(F_2_)/dT *vs.* temperature graph, it is derived from the mean of four positive cDNA samples

Among the 90 breast cancer tissues investigated, variant A was detected in 30 tumor cDNA samples (33 %) and variant E in 62 (69 %). Characteristic amplication plots of tumor cDNA samples for *COL11A1* variants A and E are shown in Figs. 6 and 7. In 28 samples, both A and E variants were detected (31 %) while in 26 samples, no variant was detected (29 %). For variant A, the mean value of copies for the positive samples was 7.58 × 10^4^ copies/μg of total RNA, while the median value was 3.28 × 10^5^ copies/μg of total RNA (range 2.36 × 10^2^-6.85 × 10^5^ copies/μg of total RNA). For variant E, the mean value of copies for the positive samples was 3.56 × 10^5^ copies/μg of total RNA, while the median value was 4.97 × 10^4^ copies/μg total RNA (range 3.51 × 10^2^-3.86 × 10^6^ copies/μg of total RNA).

### *COL11A1* transcript variants expression in relation to clinicopathological features

Statistical results are shown in Tables 4, 5 and 6. In the qualitative way, a statistically significant correlation was observed between the presence of variant E and lymph nodes involvement (*p* = 0.037) and metastasis (*p* = 0.041) (Table 5). No association was detected with the other classical prognostic factors in breast cancer. When patient tumors were classified in the higher-copy number group of the 50^th^ percentile and were also positive for variant A, they showed correlation with the better prognosis lobular histopathological type (*p* = 0.042, Table 4). The two main findings in the qualitative stats, the lymph-node involvement and the metastasis for the variant E showed a trend when examined in the 25^th^ percentile subcategories: 0.058 and 0.081 respectively (data not shown).

**Table 4:** Association of *COL11A1* variant A with clinicopathological characteristics in breast cancer tissues

	*Presence or absence of variant A (qualitative)*				*Number of A copies (percentile: 50* ^*th*^ *) (quantitative)*				*Copies of variant A (for positive samples)*	
*Clinical features*	*N (%)*	*Absence n (%)*	*Presence n (%)*	*Pearson χ* ^*2*^ *or Fisher’s exact*	*Ν (%)*	*Low*	*High*	*Pearson χ* ^*2*^ *or Fisher’s exact *	*Median*	*Mann–Whitney*
				*p-value*		*n (%)*	*n (%)*	*p-value*		*p-value*
*Age Group*				0.060				1.000		0.420
< =50 years	26 (35.6)	22 (42.3)	4 (19.0)		4 (19.0)	2 (16.7)	2 (22.2)		33,246	
> 50 years	47 (64.4)	30 (57.7)	17 (81.0)		17 (81.0)	10 (83.3)	7 (77.8)		99,636	
*Tumor Size*				0.912				1.000		0.941
≤ 2.0 cm	59 (67.0)	40 (66.7)	19 (67.9)		19 (67.9)	10 (71.4)	9 (67.9)		96,274	
> 2.0 cm	29 (33.0)	20 (33.3)	9 (32.1)		9 (32.1)	4 (28.6)	5 (35.7)		38,092	
*Histopathological Type*				0.576				*0.042*		0.071
Lobular & rest	18 (20.0)	13 (21.7)	5 (16.7)		5 (16.7)	0 (0.0)	5 (33.3)		108,055	
Intraductal infiltrating	72 (80.0)	47 (78.3)	25 (83.3)		25 (83.3)	15 (100.0)	10 (66.7)		69,402	
*Lymph-node Involvement*				0.825				0.683		0.500
Negative (Ν_0_)	55 (67.1)	38 (67.9)	17 (65.4)		17 (65.4)	7 (58.3)	10 (71.4)		106,233	
Positive (Ν_+_)	27 (32.9)	18 (32.1)	9 (34.6)		9 (34.6)	5 (41.7)	4 (28.6)		37,995	
*Metastasis*				0.124				1.000		0.570
Negative M0	47 (85.5)	32 (91.4)	15 (75.0)		15 (75.0)	7 (70.0)	8 (80.0)		96,198	
Positive M1	8 (14.5)	3 (8.6)	5 (25.0)		5 (25.0)	3 (30.0)	2 (20.0)		78,617	
*Grade*				0.352				0.683		0.328
Low (1–2)	60 (73.2)	42 (76.4)	18 (66.7)		18 (66.7)	9 (60.0)	9 (75.0)		87,999	
High (3)	22 (26.8)	13 (23.6)	9 (33.3)		9 (33.3)	6 (40.0)	3 (25.0)		30,156	
*Estrogen-receptor Status*				0.269				0.109		0.059
Negative	21 (23.9)	12 (20.3)	9 (31.0)		9 (31.0)	7 (46.7)	2 (14.3)		20,319	
Positive	67 (76.1)	47 (79.7)	20 (69.0)		20 (69.0)	8 (53.3)	12 (85.7)		100,602	
*Progesterone-receptor Status*				0.403				1.000		0.825
Negative	46 (52.3)	29 (49.2)	17 (58.6)		17 (58.6)	9 (60.0)	8 (57.1)		89,068	
Positive	42 (47.7)	30 (50.8)	12 (41.4)		12 (41.4)	6 (40.0)	6 (42.9)		56,729	
*HER2 Overexpression Status*				0.257				1.000		0.254
Negative	72 (81.8)	51 (85.0)	21 (75.0)		21 (75.0)	10 (71.4)	11 (78.6)		93,872	
Positive	16 (18.2)	9 (15.0)	7 (25.0)		7 (25.0)	4 (28.6)	3 (21.4)		31,872	
*TNBC, n (%)*				0.819				0.169		0.146
Yes	17 (19.3)	11 (18.6)	6 (20.7)		6 (20.3)	5 (33.3)	1 (7.1)		90,085	
No	71 (80.7)	48 (81.4)	23 (79.3)		23 (79.3)	10 (66.7)	13 (92.9)		20,493

**Table 5:** Association of *COL11A1* variant E with clinicopathological characteristics in breast cancer tissues

	*Presence or absence of variant E (qualitative)*				*Number of E copies (percentile: 50* ^*th*^ *) (quantitative)*				*Copies of variant E (for positive samples)*	
*Clinical features*	*N (%)*	*Absence*	*Presence*	*Pearson χ* ^*2*^ *or Fisher’s exact*	*Ν (%)*	*Low*	*High*	*Pearson χ* ^*2*^ *or Fisher’s exact*	*Median*	*Mann–Whitney*
		*n (%)*	*n (%)*	*p-value*		*n (%)*	*n (%)*	*p-value*		*p-value*
*Age Group*				0.450				0.478		0.639
< =50 years	26 (35.6)	10 (41.7)	16 (32.7)		16 (32.7)	9 (37.5)	7 (28.0)		579,678	
> 50 years	47 (64.4)	14 (58.3)	33 (67.3)		33 (67.3)	15 (62.5)	18 (72.0)		351,707	
*Tumor Size*				0.912				0.273		0.875
≤ 2.0 cm	59 (67.0)	19 (67.9)	40 (66.7)		40 (66.7)	22 (73.3)	18 (60.0)		493,555	
> 2.0 cm	29 (33.0)	9 (32.1)	20 (33.3)		20 (33.3)	8 (26.7)	12 (40.0)		106,231	
*Histopathological Type*				0.820				0.520		0.498
Lobular & rest	18 (20.0)	6 (21.4)	12 (19.4)		12 (19.4)	7 (22.6)	5 (16.1)		372,702	
Intraductal infiltrating	72 (80.0)	22 (78.6)	50 (80.6)		50 (80.6)	24 (77.4)	26 (83.9)		352,048	
*Lymph-node Involvement*				*0.037*				0.435		0.130
Negative (Ν_0_)	55 (67.1)	23 (82.1)	32 (59.3)		32 (59.3)	14 (53.8)	18 (64.3)		550,330	
Positive (Ν_+_)	27 (32.9)	5 (17.9)	22 (40.7)		22 (40.7)	12 (46.2)	10 (35.7)		145,674	
*Metastasis*				*0.041*				1.000		0.307
Negative M0	47 (85.5)	20 (100.0)	27 (77.1)		27 (77.1)	15 (78.9)	12 (75.0)		259,540	
Positive M1	8 (14.5)	0 (0.0)	8 (22.9)		8 (22.9)	4 (21.1)	4 (25.0)		467,861	
*Grade*				0.601				0.237		0.650
Low (1–2)	60 (73.2)	20 (76.9)	40 (71.4)		40 (71.4)	18 (64.3)	22 (78.6)		401,804	
High (3)	22 (26.8)	6 (23.1)	16 (28.6)		16 (28.6)	10 (35.7)	6 (21.4)		119,249	
*Estrogen-receptor Status*				0.367				0.876		0.867
Negative	21 (23.9)	5 (17.9)	16 (26.7)		16 (26.7)	8 (25.8)	8 (27.6)		228,414	
Positive	67 (76.1)	23 (82.1)	44 (73.3)		44 (73.3)	23 (74.2)	21 (72.4)		411,925	
*Progesterone-receptor Status*				0.453				0.287		0.174
Negative	46 (52.3)	13 (46.4)	33 (55.0)		33 (55.0)	15 (48.4)	18 (62.1)		305,909	
Positive	42 (47.7)	15 (53.6)	27 (45.0)		27 (45.0)	16 (51.6)	11 (37.9)		432,753	
*HER2 Overexpression Status*				0.517				1.000		0.592
Negative	72 (81.8)	24 (85.7)	48 (80.0)		48 (80.0)	24 (80.0)	24 (80.0)		430,475	
Positive	16 (18.2)	4 (14.3)	12 (20.0)		12 (20.0)	6 (20.0)	6 (20.0)		104,271	
*TNBC, n (%)*				0.732				0.100		0.146
Yes	17 (19.3)	6 (21.4)	11 (18.3)		11 (18.3)	3 (9.7)	8 (27.6)		323,558	
No	71 (80.7)	22 (78.6)	49 (81.7)		49 (81.7)	28 (90.3)	21 (72.4)		371,840

**Table 6:** Association of both or either *COL11A1* A and E variants with clinicopathological characteristics in breast cancer tissues

*Clinical features*	*Ν (%)*	*Both Variant A & E*	*Rest*	*Pearson χ* ^*2*^ *or Fisher’s exact*	*N (%)*	*Either Variant A OR E*	*No variant*	*Pearson χ* ^*2*^ *or Fisher’s exact*
		*n (%)*	*n (%)*	*p-value*		*n (%)*	*n (%)*	*p-value*
*Age Group*				*0.036*				0.535
< =50 years	26 (35.6)	3 (15.8)	23 (42.6)		26 (35.6)	17 (33.3)	9 (40.9)	
> 50 years	47 (64.4)	16 (84.2)	31 (57.4)		47 (64.4)	34 (66.7)	13 (59.1)	
*Tumor Size*				0.778				0.778
≤ 2.0 cm	59 (67.0)	18 (69.2)	41 (66.1)		59 (67.0)	41 (66.1)	18 (69.2)	
> 2.0 cm	29 (33.0)	8 (30.8)	21 (33.9)		29 (33.0)	21 (33.9)	8 (30.8)	
*Histopathological Type*				0.733				0.642
Lobular & rest	18 (20.0)	5 (17.9)	13 (21.0)		18 (20.0)	12 (18.8)	6 (23.1)	
Intraductal infiltrating	72 (80.0)	23 (82.1)	49 (79.0)		72 (80.0)	52 (81.3)	20 (76.9)	
*Lymph-node Involvement*				0.571				0.072
Negative (Ν_0_)	55 (67.1)	15 (62.5)	40 (69.0)		55 (67.1)	34 (60.7)	21 (80.8)	
Positive (Ν_+_)	27 (32.9)	9 (37.5)	18 (31.0)		27 (32.9)	22 (39.3)	5 (19.2)	
*Metastasis*				0.098				*0.043*
Negative M0	47 (85.5)	13 (72.2)	34 (91.9)		47 (85.5)	29 (78.4)	18 (100.0)	
Positive M1	8 (14.5)	5 (27.8)	3 (8.1)		8 (14.5)	8 (21.6)	0 (0.0)	
*Grade*				0.215				0.810
Low (1–2)	60 (73.2)	16 (64.0)	44 (77.2)		60 (73.2)	42 (72.4)	18 (75.0)	
High (3)	22 (26.8)	9 (36.0)	13 (22.8)		22 (26.8)	16 (27.6)	6 (25.0)	
*Estrogen-receptor Status*				0.166				0.509
Negative	21 (23.9)	9 (33.3)	12 (19.7)		21 (23.9)	16 (25.8)	5 (19.2)	
Positive	67 (76.1)	18 (66.7)	49 (80.3)		67 (76.1)	46 (74.2)	21 (80.8)	
*Progesterone-receptor Status*				0.383				0.457
Negative	46 (52.3)	16 (59.3)	30 (49.2)		46 (52.3)	34 (54.8)	12 (46.2)	
Positive	42 (47.7)	11 (40.7)	31 (50.8)		42 (47.7)	28 (45.2)	14 (53.8)	
*HER2 Overexpression Status*				0.546				0.375
Negative	72 (81.8)	20 (76.9)	52 (83.9)		72 (81.8)	49 (79.0)	23 (88.5)	
Positive	16 (18.2)	6 (23.1)	10 (16.1)		16 (18.2)	13 (21.0)	3 (11.5)	
*TNBC, n (%)*				0.646				0.563
Yes	17 (19.3)	6 (22.2)	11 (18.0)		17 (19.3)	11 (17.7)	6 (23.1)	
No	71 (80.7)	21 (77.8)	50 (82.0)		71 (80.7)	51 (82.3)	20 (76.9)	

**Fig. 8:**
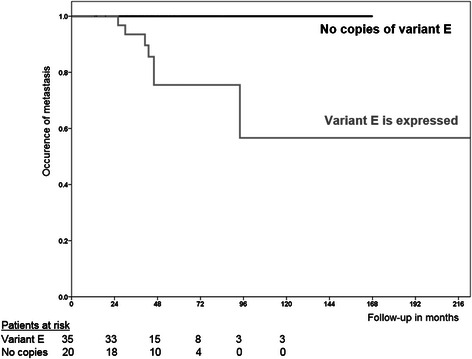
Figure 8 Kaplan-Meier survival analysis with respect to long-term metastasis in 55 of 90 breast cancer patients (35 patients with variant E expression vs. 20 without variant E expression), where follow-up data was available

When examining the simultaneous expression of variant A and variant E, that was significantly correlated with the older age group (*p* = 0.036, Table 6 left). Furthermore, the qualitative presence of either variant A or either variant E presented a significant correlation with metastasis (*p* = 0.043, Table 6 right). There was also a statistically significant positive correlation between copies of variant A and copies of variant E (rho = 0.368, *p* = 0.050). We also examined the association of *COL11A1* transcript variants with metastasis in the 55 patients where follow-up data was available by using the Kaplan-Meier survival analysis. Patients with the presence of variant E in their tumor showed a reduced disease-free interval compared to those not expressing it (*p* = 0.060, log-rank test, Fig. 8).

## Discussion

The first goal of this study was the development and validation of new and reliable quantitative assays for all reported *COL11A1* mRNA splice variants (A, B, C and E) by using real-time qPCR methods. With another simple conventional PCR technique -in a common genomic area for all transcripts- we would still being able to determine the presence or not of the *COL11A1* gene transcript, in general. Furthermore, we applied these techniques in breast cancer tissues in order to use the obtained quantitative data to determine any existing significant correlation between the differential expression of *COL11A1* variants and clinicopathological features of these patients.

When 90 breast cancer tissues were studied, only A and E variants were encountered while the general *COL11A1* transcript was present in all samples. Variant A was detected in 30 samples (33 %) and variant E in 62 (69 %). In 28 samples, both A and E variants were detected (31 %) while in 26 samples, no variant was detected (29 %). Variants B and C were not detected in our series of samples and hence, we were not able to validate the methodologies with the proposed combination of primers and probes. The quantification of variants A and E was performed with a real-time qPCR methodology on the LightCycler 1.5 thermocycler using dual hybridization probes and melting curve analysis at the end of each reaction. We performed optimization experiments by using isolated and quantified amplicons as external standards of the developed real-time qPCR assays for the A and E variants. The assays were developed satisfactorily, were rapid and reliable, demonstrating excellent efficiencies (2.05 ± 0.04 for variant A and 1.88 ± 0.10 variant E), very good reproducibilities (CV ≤1.3 % for variant A and CV ≤3.2 % for variant E) and low detection limits (~19 copies/μL for variant A and ~16 copies /μL for variant E). The specificity of the real-time qPCR assays was tested by melting curve analysis (T_m_ of variant A amplicon was 69.9 (±1.0) °C while that of variant E was 65.3 (±1.0) °C), by the presence of specific bands of the proper size during electrophoresis of the real-time PCR products and finally, by DNA sequencing of the amplicons obtained. The determination was easy and rapid (within ~ 50 min) after the synthesis of the cDNA and it was possible to analyze up to 32 samples simultaneously. However, there is the possibility of higher throughput in larger platforms such as the LightCycler 480/1630, wherein the determinations that are performed in microtiter plates lead to a much greater number of samples that can be processed together.

Statistical analysis of the data was carried out in order to detect any existing significant correlation between the differential expression of the variants A and E (presence or not, low or high number of copies) with clinicopathological characteristics of the samples and the patients (such as age group, tumor size, histopathological type of tumor, lymph nodes involvement, grade, metastasis, hormone receptors status, *HER2* oncogene overexpression, TNBC status). The copy numbers of variants A and were E showed some positive correlation between them (rho = 0.368, *p* = 0.050) and the simultaneous expression of them was significantly correlated with the older age group (*p* = 0.036). We cannot exclude that this might reflect a more generalized defect in the splicing machinery with increased aging. The most important finding was the observed statistically significant correlation between the presence of variant E and lymph nodes involvement (*p* = 0.037) and metastasis (*p* = 0.041) which was corroborated by a trend in Kaplan-Meir analysis where the patients with variant E in their tissue show reduced disease-free interval (*p* = 0.060). Furthermore, the qualitative presence of either variant A or variant E showed a significant correlation with metastasis (*p* = 0.043). Results could be probably reinforced if follow-up data was available for all 90 patients with quantitative data on variants A and E and not only for 55 patients. No other association with established histopathological prognostic parameters was detected in our results. A working hypothesis therefore, would be that the shorter isoform, produced from the translation of variant E mRNA, would be more resistant in proteolytic actions by enzymes such as BMP-1 [27]- and it could retain the bulky NTD domain for a longer time. This could lead to a “thinner” collagenous stroma, more attractive to adhesion molecules and metalloproteinases (as NTD contains thrombospondin-1 like and heparin binding regions [39]) and thus, could pave the way for tumor cells motility and metastasis.

A limitation of our study is that we could not investigate quantitatively whether the breast tumor cells showed upregulation of the expression of variants compared to normal epithelial breast tissues. Also, we could not dissect the expression to either the epithelial or the stromal compartment as the specimens obtained were a mixture of these. Finally, regarding the group of breast tumor tissues examined, the tumors studied were relatively small (~2.0 cm) because they originated from well-monitored patients in a metropolitan area. During the total RNA isolation procedure, although the samples were placed directly into an appropriate material for the RNA stability (RNAlater), the presence of inhibitors in our fresh-frozen biopsy RNA preparations and their integrity were not assessed by assays such as the SPUD [40] and the 5:3 ratio GAPDH (GlycerAldehyde 3-Phosphate DeHydrogenase) mRNA integrity tests [36]. However, the RNA quality was tested with the actin reference gene and measured with absolute accuracy with the Quant-It RNA Assay kit on Qubit. Differences in cDNA synthesis efficiency due to tumor variability could not be assessed since the absolute quantification and normalization to total RNA strategy was selected for analysis of data (and not relative quantification and normalization to expression of one or an average of three reference genes as is the trend nowadays).

## Conclusions

This study was the first to assess the differential expression of *COL11A1* A and E splice variants in breast cancer tissues and in cancer in general. We attempted also to detect B and C variants but with no clear indication whether our assays failed or these transcripts weren’t present, since we didn’t possess any positive control. The existence of other variants is speculated: the fact that in 29 % of the cDNA samples no *COL11A1* variant were detected -despite the presence of the general transcript- warrants a new research effort in the future for the quest and identification of novel variants. Additionally, the general *COL11A1* transcript could also be quantitated in a novel assay (e.g. multiplexed with A and/or E variants) in order to identify samples that although they are positive for A and/or E variants don’t sum up to the total *COL11A1* transcript and therefore one could hypothesize that they contain additional aberrant transcripts.

The study also could be extended to a larger number of breast cancer tissues and a significant number of normal tissues so that it could verify the results of earlier studies in relation to increased or no expression of *COL11A1* mRNA and its variants in breast cancer. In this case, it may be possible to include *COL11A1* gene and/or its variants in new improved prognostic multiparameter expression arrays for predicting metastasis. This information would be useful for 20–30 % of lymph node positive breast cancer patients that remain free of distant metastasis in 15–30 years but still receive toxic chemotherapy [22]. It is expected that new tools such as deep RNA Sequencing with Next Generation Sequencing (NGS) platforms could assist in the discovery of such new aberrant transcripts in tumor RNA samples.

By employing polyclonal antibodies against various epitopes in the NTD domain -that are available now at a research level [21, 41]-, it should be possible to further validate our assays of *COL11A1* RNA variants and to evaluate findings on the differential proteolysis of the N-terminal regions of the protein chain of collagen a1(XI) in breast cancer and their involvement in tissue remodeling through stereochemistry. The combined use of laboratory tools such as qPCR and Western Blot would lead to validation of antibodies suitable for use in routine IHC in paraffin-embedded tissues. Also it would be useful to evaluate the expression of *COL11A1* variants in other cancers such as oropharynx [17], ovarian [18] and lung cancer [15], wherein the expression of *COL11A1* has been shown to be associated with disease progression.

## Additional files

Additional file 1: Figure S1. Conventional PCR products for the general *COL11A1* transcript run on a 2 % w/v agarose gel: in lane 1 PCR MW Marker (50-150-300-500-766 bp), lanes 2–5 positive cDNA samples for the general transcript (132 bp), lane 6 blank. (JPEG 43 kb)
Additional file 2: Figure S2. PCR products from inverted capillaries of positive tumor samples for *COL11A1* splice variant A run on a 2 % w/v agarose gel: in lane 1 PCR MW Marker (50-150-300-500-766 bp), lane 2 blank, lanes 3–7 positive cDNA samples (439 bp). (JPEG 13 kb)
Additional file 3: Figure S3. PCR products from inverted capillaries of positive tumor samples for *COL11A1* splice variant E run on a 2 % w/v agarose gel: in lane 1 PCR MW Marker (50-150-300-500-766 bp), lane 2 blank, lanes 3–7 positive cDNA samples (259 bp). (JPEG 15 kb)
Additional file 4: Figure S4. Sanger DNA Sequencing electropherogram from a positive amplicon for *COL11A1* transcript variant A in a tumor cDNA sample. (JPEG 133 kb)
Additional file 5: Figure S5. Sanger DNA Sequencing electropherogram from a positive amplicon for *COL11A1* transcript variant E in a tumor cDNA sample. (JPEG 125 kb)

## Abbreviations

CISH: Chromogenic in situ hybridization
Cq: Quantification cycle
CV: Coefficient of variation
ECM: Extracellular matrix
EMT: Epithelial-to-mesenchymal transition
IHC: Immunohistochemistry
NTD: N-terminal domain
qPCR: Quantitative Polymerase Chain Reaction
Tm: Melting point temperature
TNBC: Triple negative breast cancer
