# Supplementary figures and images for: Development of novel real-time PCR methodology for quantification of COL11A1 mRNA variants and evaluation in breast cancer tissue specimens
Source: BMC Cancer. 2015 Oct 14;15:694. doi: 10.1186/s12885-015-1725-8 (PMC4606509; doi:10.1186/s12885-015-1725-8)

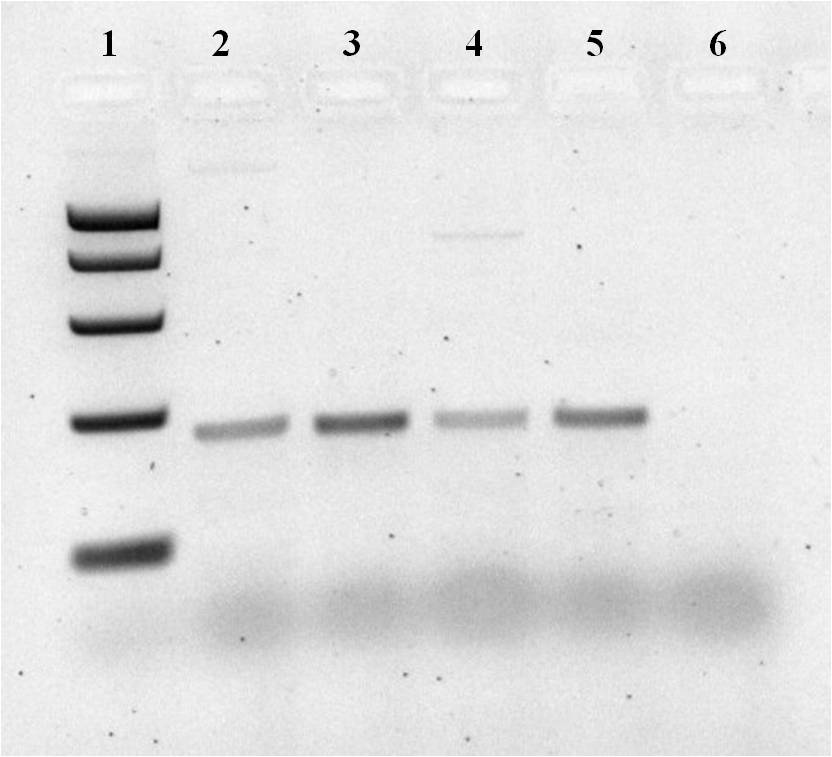

Supplement: Additional file 1: Figure S1. — Conventional PCR products for the general COL11A1 transcript run on a 2 % w/v agarose gel: in lane 1 PCR MW Marker (50-150-300-500-766 bp), lanes 2–5 positive cDNA samples for the general transcript (132 bp), lane 6 blank. (JPEG 43 kb) [file 12885_2015_1725_MOESM1_ESM.jpeg]

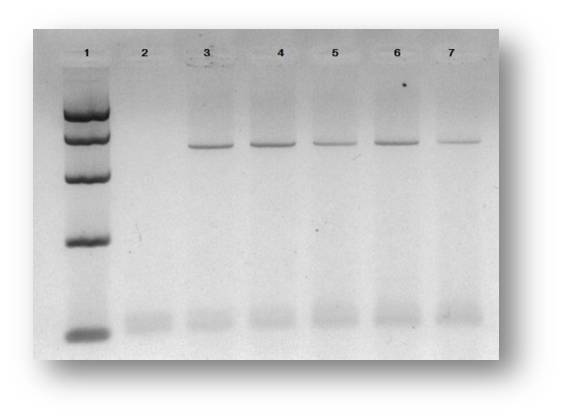

Supplement: Additional file 2: Figure S2. — PCR products from inverted capillaries of positive tumor samples for COL11A1 splice variant A run on a 2 % w/v agarose gel: in lane 1 PCR MW Marker (50-150-300-500-766 bp), lane 2 blank, lanes 3–7 positive cDNA samples (439 bp). (JPEG 13 kb) [file 12885_2015_1725_MOESM2_ESM.jpeg]

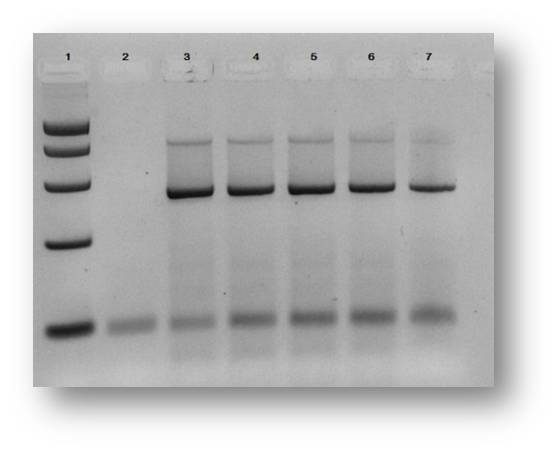

Supplement: Additional file 3: Figure S3. — PCR products from inverted capillaries of positive tumor samples for COL11A1 splice variant E run on a 2 % w/v agarose gel: in lane 1 PCR MW Marker (50-150-300-500-766 bp), lane 2 blank, lanes 3–7 positive cDNA samples (259 bp). (JPEG 15 kb) [file 12885_2015_1725_MOESM3_ESM.jpeg]

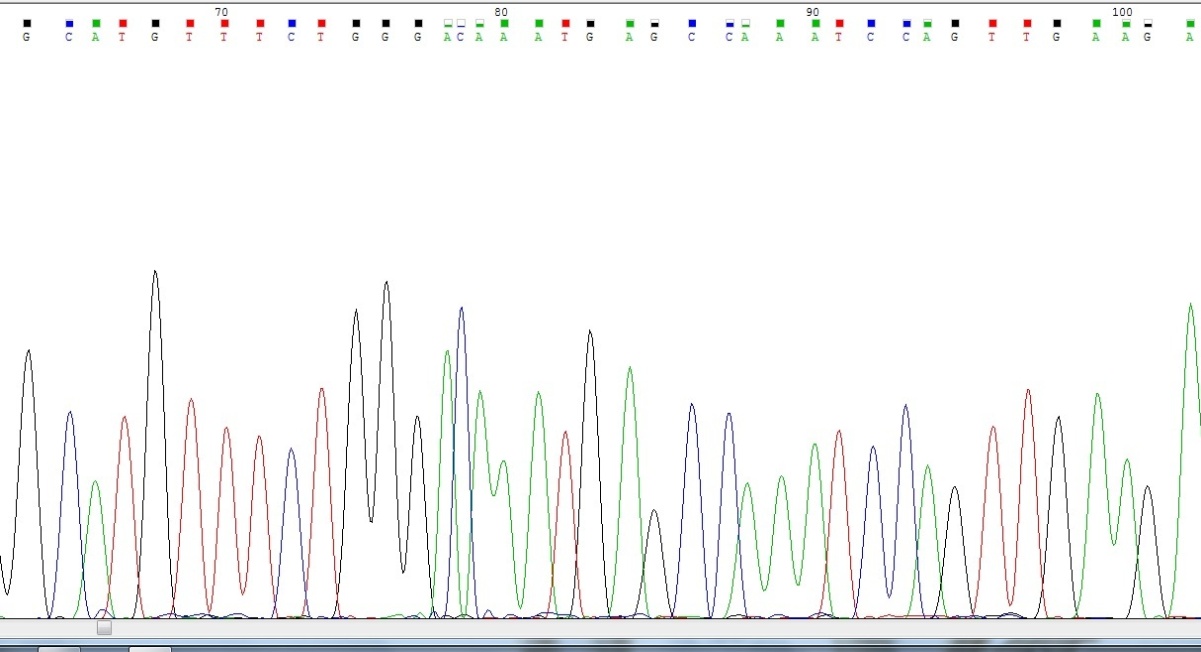

Supplement: Additional file 4: Figure S4. — Sanger DNA Sequencing electropherogram from a positive amplicon for COL11A1 transcript variant A in a tumor cDNA sample. (JPEG 133 kb) [file 12885_2015_1725_MOESM4_ESM.jpeg]

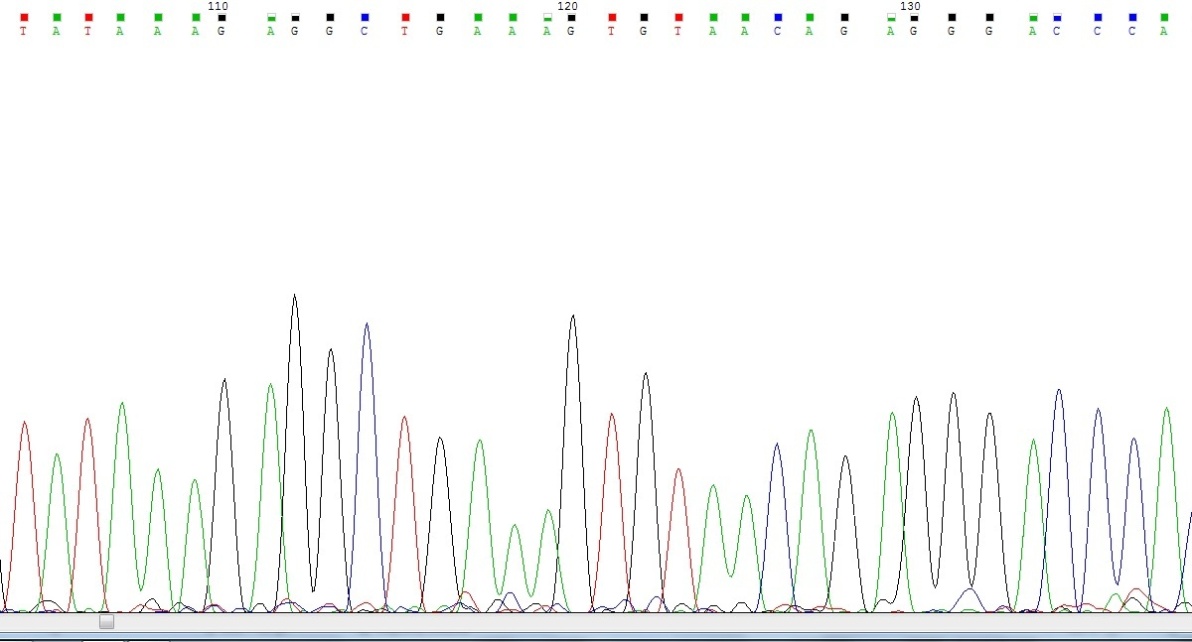

Supplement: Additional file 5: Figure S5. — Sanger DNA Sequencing electropherogram from a positive amplicon for COL11A1 transcript variant E in a tumor cDNA sample. (JPEG 125 kb) [file 12885_2015_1725_MOESM5_ESM.jpeg]
